# Seed Priming With Melatonin Promotes Seed Germination and Seedling Growth of *Triticale hexaploide* L. Under PEG-6000 Induced Drought Stress

**DOI:** 10.3389/fpls.2022.932912

**Published:** 2022-06-30

**Authors:** Yuhui Guo, Dongxiao Li, Liantao Liu, Hongchun Sun, Lingxiao Zhu, Ke Zhang, Haiming Zhao, Yongjiang Zhang, Anchang Li, Zhiying Bai, Liwen Tian, Hezhong Dong, Cundong Li

**Affiliations:** ^1^State Key Laboratory of North China Crop Improvement and Regulation/State Key Laboratory of Cotton Biology (Hebei Base)/Key Laboratory of Crop Growth Regulation of Hebei Province/College of Agronomy, Hebei Agricultural University, Baoding, China; ^2^Cotton Research Center, Shandong Key Lab for Cotton Culture and Physiology, Shandong Academy of Agricultural Sciences, Jinan, China; ^3^Institute of Industrial Crops, Xinjiang Academy of Agricultural Sciences, Urumqi, China; ^4^Institute of Dryland Farming, Hebei Academy of Agriculture and Forestry Sciences, Key Laboratory of Crop Drought Resistance Research of Hebei Province, Hengshui, China

**Keywords:** drought stress, melatonin, triticale, germination, seedling, antioxidative activity

## Abstract

Melatonin (N-acetyl-5-methoxytryptamine, MT) can mitigate abotic stress, including drought stress on a number of crops. However, it is unclear whether and how seed priming with melatonin alleviates the effects of drought stress on seed germination and seedling growth of triticale (*Triticale hexaploide* L.). In this study, we investigated the effects of seed priming with MT on seed germination, protective enzyme activity, superoxide anion, and hydrogen peroxide in triticale under PEG-6000 induced drought stress. Seed priming with 20 μM MT alleviated the adverse effects of PEG-6000 induced drought stress on seed germination and seedling growth. Triticale seeds primed with 20 μM MT exhibited improved germination potential, germination rate, germ and radicle length. Specifically, MT priming increased the germination rate by 57.67% compared with unprimed seeds. Seed priming with melatonin also alleviated the adverse effects of PEG-6000 induced drought stress on triticale seedlings. MT pretreatment with 20 μM significantly increased the net photosynthetic rate, transpiration rate, stomatal conductance, plant height, leaf area, and relative chlorophyll concentration, enhanced the activities of superoxide dismutase and peroxidase, and decreased reactive oxygen species (ROS) and malonaldehyde content in the seeds (germ and radicle) and seedlings (leaf and root). Collectively, these results suggest that seed priming with melatonin promotes ROS scavenging capacity and enhances energy supply and antioxidant enzyme activities to alleviate the adverse effects of drought stress in triticale.

## Introduction

Triticale (*Triticale hexaploide* L.) is a new crop species bred from wheat (*Triticum aestivum* L.) and rye (*Secale cereal* L.) through intergeneric hybridization and doubling of hybrid chromosomes (Ayalew et al., 2018). Triticale is emerging as an important source of food and feed owing to its good nutritional quality, strong stress resistance, wide adaptability, and high yield. Triticale is popularly used as livestock feed because of its high crude protein content, fresh and juicy stalk, good palatability, and high digestibility (Royo and Pares, 2010). The increase in development of animal husbandry has accelerated the planting of triticale as a high-quality forage grass. The current cultivation area of triticale in China is 195,000 hm^2^, making it one of the most cultivated forage crops in China (FOASTAT, 2022).

Global climate change has increased the risk of short-term extreme climate stresses in agriculture. Drought is emerging as one of the most significant abiotic stresses limiting crop growth and development (Lafitte et al., 2007; Cruz de Carvalho, 2008). Drought stress severely affects seed germination and seedling establishment, which is detrimental to the establishment of high-yielding population, and ultimately leads to poor yield (Sun et al., 2015). Drought is also a major environmental limiting factor for triticale seed germination and seedling development (Liu et al., 2015b). Therefore, it is important to explore effective ways of alleviating the effects of drought stress on field-grown triticale.

Seed priming is an important method for alleviating abiotic stress in crops. It improves the emergence and growth rate of seedlings, shortens the growth period of crops, and improves productivity (Nawaz et al., 2013). Seed priming enhances seed germination and seedling development by maintaining the structure of the seed (protects membrane during imbibition) and refining genetic repair, RNA and protein synthesis, and antioxidant mechanisms (Saddiq et al., 2019). Previous studies in cotton and maize (Guan et al., 2009; Singh et al., 2018) revealed that priming can significantly promote seed germination and seedling growth, and improve germination performance and seedling establishment under abiotic stress (Ellouzi et al., 2021). Many seed initiation methods have been established, including hormone priming, osmopriming, hydropriming, stratification, humidification, hardening, and thermal shock (Nawaz et al., 2017). Common initiators are polyethylene glycol (PEG), salicylic acid (SA), abscisic acid (ABA), and gibberellic acid (GA3) (Jisha et al., 2013). Hormone priming is an efficient and cost-effective method, but is affected by several factors, such as plant species, seed viability, initiator concentration, and initiation method (Nawaz et al., 2017). Therefore, it is crucial to determine the most effective seed priming agent and concentration to achieve the desired results.

Melatonin (N-acetyl-5-methoxytryptamine, MT) was first discovered in plants in 1995. Prior to its discovery, melatonin was thought to be a hormone unique to animals (Dubbels et al., 1995). MT is a regulatory molecule with multiple biological functions, including modulating seed germination, root development, leaf senescence, and fruit ripening in plants (Ahammed et al., 2020). MT also plays a vital role in abiotic stress responses in plants (Arnao and Hernández-Ruiz, 2014; Zhang et al., 2015). Studies have found that exogenous MT can alleviate oxidative damage caused by drought stress in wheat (Cui et al., 2017) and maize (Huang et al., 2019), and promote seed germination and seedling growth of various crops (Liang et al., 2019). Melatonin-priming enhanced the tolerance of upland cotton to drought stress by modulating the antioxidant system (Supriya et al., 2022). Khan et al. also found that priming rape seeds with MT could remove excess reactive oxygen species by improving the activity of antioxidant enzymes to prevent chloroplast self-digestion and cell-wall degradation (Khan et al., 2020). This alleviates drought stress-induced inhibition of seed germination. Antoniou et al. (2017) also demonstrated that MT priming can improve drought tolerance of alfalfa plants by regulating homeostasis of nitro-oxidation and osmotic protection.

MT priming has a positive effect on improving stress resistance of crops however, little is known about its effects on the germination, seedling development, physiology, and epidermal microstructure of triticale seeds under drought stress. Therefore, we investigated the effect of MT priming on triticale seed germination and seedling development and its physiological effects under drought stress conditions. This study aimed to (1) screen the optimum MT concentration for seed priming, (2) characterize the morphology of seedlings and changes in protective enzyme system, and (3) explore the drought-response mechanism underlying the morphology, physiology, and antioxidant enzyme systems of seedlings.

## Materials and Methods

### Materials

Triticale seeds (cultivar Zhongsi-1048) were obtained from Dryland Farming Institute, Hebei Academy of Agriculture and Forestry Sciences. Melatonin was purchased from SIGMA Corp., USA. Assay kits for determining activities/contents of malonaldehyde (MDA), superoxide dismutase (SOD), peroxidase (POD), hydrogen peroxide (H_2_O_2_) and total protein (TP) were purchased from Nanjing Jiancheng Bioengineering Institute (China). Assay kit for determining superoxide anion (O_2_^−^) content was purchased from Beijing Solarbio Technology Com. Ltd., China.

### Experiment 1

#### Screening for Optimum MT Priming Concentration

The experiment was conducted in August of 2021 in the laboratory of crop growth regulation at Hebei Agricultural University, Baoding (38.85°N, 115.30°E), Hebei Province, China. The triticale seeds were surface sterilized using 5% (v/v) NaClO for 10 min, then washed three times with sterile distilled water to remove disinfectant residues. The disinfected seeds were transferred into beakers containing different concentrations of MT solution (0, 1, 10, 20, 50, 100, and 200 μM). The ratio of seeds to MT solution was 1:5 (w/v). The beakers were then transferred into an incubator set at 15 °C, and incubated for 24 h without light. The MT solutions were renewed every 12 h (Zheng et al., 2016). After priming, the seeds were rinsed three times with distilled water to remove MT residue, and then dried for 48 h at 25 °C. Afterward, the triticale seeds were stored at 4 °C in a refrigerator for later use.

A total of 25 seeds from each treatment were placed on separate 150 ml Petri dishes with three layers of germination paper. Subsequently, 10 ml of 10% PEG-6000 treatment solution was added to each Petri dish, which was then placed in an incubator set at 25 °C. The 10% PEG-6000 treatment solution was supplemented every 48 h to 72 h. Each treatment was replicated five times. The germination rate was calculated to determine the optimal MT concentration for seed priming.

### Experiment 2

#### Seed Priming With Melatonin Under Drought Stress

##### Petri Dish Experiment

Four treatments with six replicates each were used. The treatments were as follows: no seed priming + water (CK), no seed priming +10% PEG-6000 (DS; drought stress), 20 μM MT priming + water (MT), 20 μM MT priming +10% PEG-6000 (MT+DS). The water potential of 10% PEG-6000 was −2.18 MPa for inducing drought in seedlings. For determination of the optimal MT priming concentration, fifty seeds were placed in each petri dish for each treatment and incubated at 25 °C in darkness. The length and fresh weight of germ and radicle were recorded on the 7^th^ day. The dry matter of germ and radicle were weighed separately after the materials were dried at 60 °C to a constant weight. The number of germinated seeds was recorded daily for 7 days. The measurements were conducted according to the International Rules for Seed ISTA Testing. The germination potential (GP), germination rate (GR), germination index (GI), and vigor index (VI) of the triticale seeds were calculated according to the following formulas (Hatami et al., 2014):


$$\begin{array}{l} \mathrm{GP(}\%)=\mathrm{Number of normally germinated seeds}\;\left(\mathrm{on}\;{\mathrm{the 3}}^{\mathrm{rd}}\;\mathrm{day}\right)/\\ \;\;\mathrm{Number of seeds tested}\times 100. \end{array}$$

$$\begin{array}{l} \mathrm{GR(}\%)=\mathrm{Total number of normally germinated seeds}/\\ \;\;\mathrm{Number of seeds tested}\times 100. \end{array}$$

$$\mathrm{GI}=\sum \mathrm{Gt}/\mathrm{Dt}\;\left(\begin{array}{l} \mathrm{Gt}\;\mathrm{is the}\;\mathrm{GR}\;\mathrm{corresponding to}\;\mathrm{Dt, and}\;\\ \mathrm{Dt}\;\mathrm{is the}\;\mathrm{day}\;\mathrm{of the germination test} \end{array}\right).$$

VI=GI×W(Wis the fresh weight of germinated seeds).

##### Pot Experiment

Sterilized seeds were sown in a cylindrical culture pot (9 cm in height and 13 cm in diameter; ten seeds per pot) containing 700 g compound culture medium (soil: nutrient soil: sand = 3:1:1) in an environmentally controlled greenhouse. The experiment had four treatment groups: no seed priming +70–75% relative water content (RWC) (CK), no seed priming +40–45% RWC (DS), 20 μM MT priming +70–75% RWC (MT), and 20 μM MT priming +40–45% RWC (MT+DS). Each treatment contained ten replicates. The relative soil moisture content was controlled from the time of seed sowing by weighing method depending on the amount of irrigation. Environmental conditions of the greenhouse were as follows: relative humidity 40–45%, temperature 25/20 °C (day/night), light/dark 16 h/8 h, and light intensity of 600 μmol·m^−2^·s^−1^. After 10 and 20 d of drought exposure, the leaves of the seedlings were harvested to determine the parameters associated with plant growth, gas exchange and physiological traits.

### Measurement of Plant Growth and Gas Exchange Parameters

The length and width of leaves and plant height (PHT) were measured using a ruler 10 and 20 days after sowing on the culture pots. Leaf area (LEA) was calculated using the length–width coefficient method. Relative chlorophyll concentrations (SPAD) of the leaves were determined using a SPAD meter (SPAD-502, Konica-Minolta, Tokyo, Japan). Gas exchange parameters, including the net photosynthetic rate (Pn), intercellular CO_2_ concentration (Ci), transpiration rate (Tr), and stomatal conductance (Gs) of the leaves were measured using a portable photosynthesis system (LI-6400, Li-COR, Lincoln, NE, USA). Twelve light intensities (2000, 1800, 1500, 1200, 1000, 800, 500, 200, 100, 50, 20, 0) were set in the light range of 2000–0 μmol/(m^2^·s), the temperature in the chamber was controlled at 25 °C, and the CO_2_ concentration was set as 400 μmol/mol. Three leaves with consistent growth in each treatment were selected, and the results were averaged. Light compensation point (LCP), light saturation point (LSP), apparent dark respiration rate (Rd), maximum net photosynthetic rate (Pn_max_), and apparent quantum efficiency (AQE) were determined by light response curve.

### Measurement of Physiological Traits

Germ and radicle were collected on the third and seventh days of the petri dish experiment. Leaves and roots were collected on the tenth and twentieth days of the pot experiment. The collected samples were used to determine the activity of SOD and POD, and the contents of MDA, TP, and H_2_O_2_ as described by the manufacturers of corresponding assay kits.

Histochemical Detection of H_2_O_2_ and O_2_^−^ in Leaves and Roots

DAB (3,3-diaminobenzidine) staining of root tips was performed to determine H_2_O_2_ accumulation. DAB reacts with H_2_O_2_ to produce a brown complex compound (Thordal-Christensen et al., 1997). Briefly, the root tips were cut (at 0–20 mm) and placed in 1% (w/v) DAB solution (DAB dissolved in 10 mM MES buffer, pH 6.5), and incubated at 25 °C for 2 h in the dark. After rinsing sufficiently, the root tip was observed and photographed using a stereomicroscope. The functional leaves of the main stem were incubated in DAB solution for 16 h in the dark. The leaves were then decolorized by boiling them in 75% alcohol and photographed using a camera (NikonD7100, Japan). The content of H_2_O_2_ was quantified by measuring the size of the leaf spots.

Nitro blue tetrazolium (NBT) chloride was used for O_2_^−^ staining of root tips to determine their O_2_^−^ contents. NBT chloride reacts with O_2_^−^ to produce a blue-violet complex compound (Dunand et al., 2007). Briefly, the root tips were cut (0–20 mm) and placed in 100 mM NBT (dissolved in 50 mM PBS buffer) solution. After staining for 5 min, the root tips were rinsed with PBS, observed, and photographed under a stereo-microscope. The leaves were incubated for 8 h in the dark at 25 °C, then boiled in 75% alcohol to decolorize. Finally, O_2_^−^ content was quantified by measuring the size of the leaf spots.

### Statistical Analysis

All the experimental data were analyzed using SPSS 26.0 (IBM, Inc., Armonk, NY, USA). The differences between various treatments were determined using one-way analysis of variance (ANOVA), followed by new multiple range (Duncan) test (*p* ≤ 0.05). Figures were drawn using GraphPad Prism 8 (GraphPad Software Inc., CA, USA).

## Results

### Optimal MT Concentration for Priming

**Figure 1:**
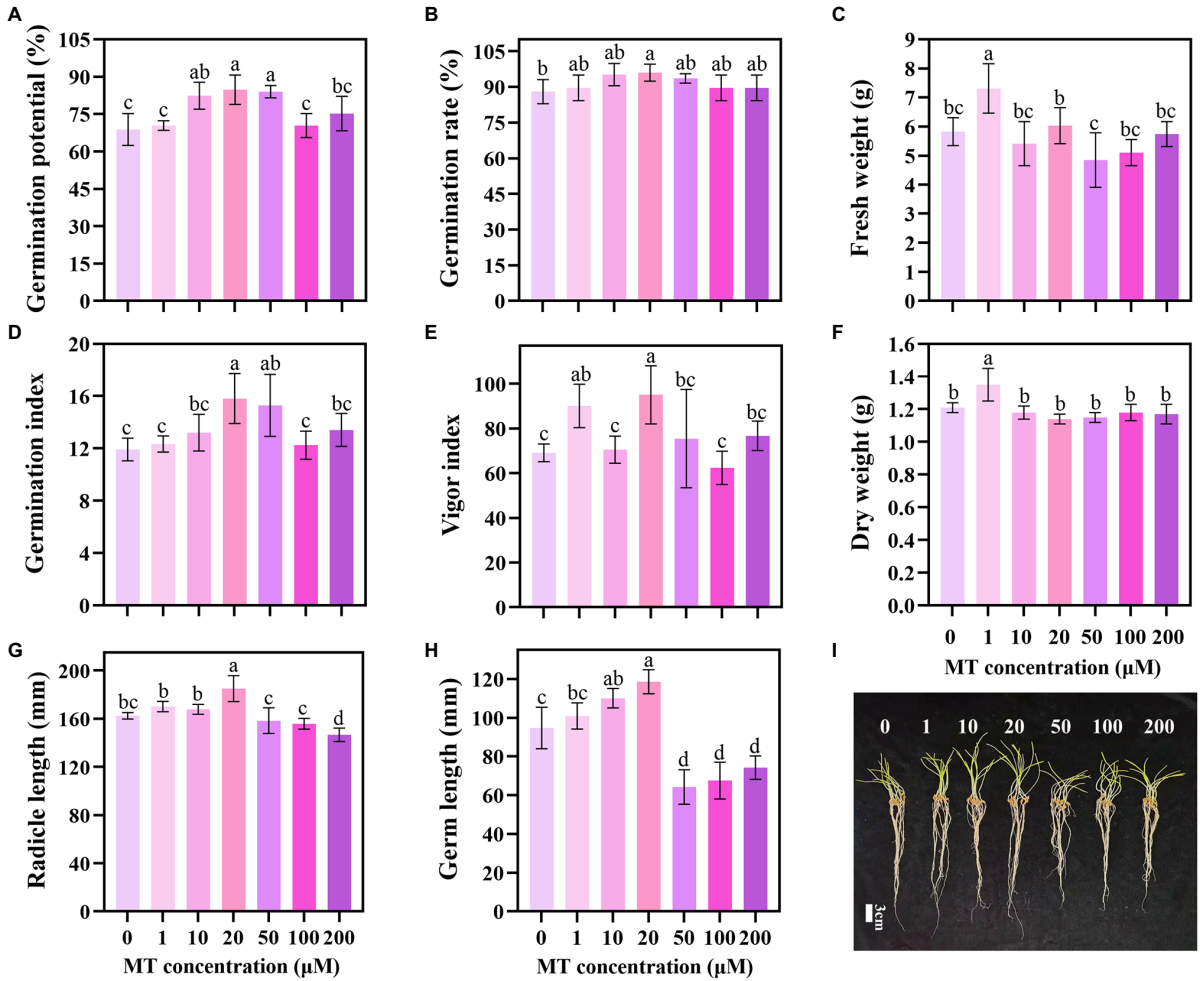
Germination characteristics of triticale seeds following treatment with different MT concentrations under drought stress. **(A)** Germination potential (GP), **(B)** germination rate (GR), **(C)** fresh weight, **(D)** germination index (GI), **(E)** vigor index (VI), **(F)** dry weight, **(G)** radicle length, **(H)** germ length, and **(I)** germination of MT primed triticale seeds after 7 days. Both **(G,H)** values are the means of 10 replicates ± standard error, while other data are the means of five replicates ± standard error. For each trait, bars with the same letter are not significantly different at *p* < 0.05. Different lower letters indicate significant differences among the treatments (*p* < 0.05).

The priming effect showed a trend of first increasing and then decreasing with the increase in MT concentration. MT concentration of 20 μM increased the GP, GR, GI, and VI of triticale seeds by 23.26, 9.09, 32.63, and 25.97%, respectively, compared with the CK (Figures 1A,B,D,E). Lower concentrations of MT (1, 10, and 20 μM) promoted the elongation of the germ and embryo of the seeds under DS, while higher concentrations (50, 100, and 200 μM) inhibited the elongation. The lengths of germ and radicle of triticale seeds primed with 20 μM MT were the longest, increasing by 25.18 and 13.96%, respectively, compared with 0 μM MT; and 17.52 and 8.72%, respectively, compared with 1 μM MT (Figures 1G–I). These results indicate that 20 μM is the optimum concentration for MT priming of triticale seeds.

### Effect of MT Priming on Seed Germination Under Drought Stress

**Figure 2:**
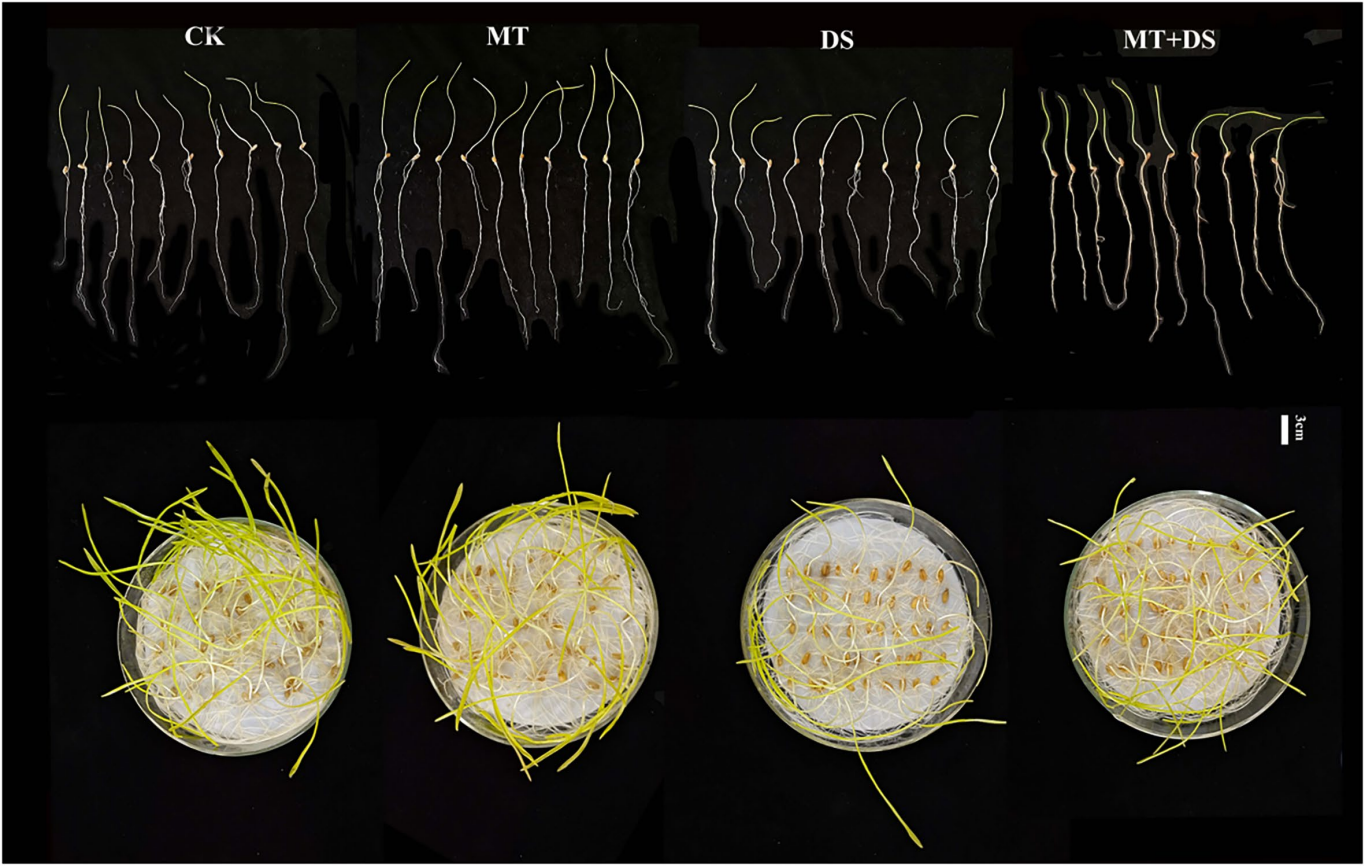
Phenotypic traits of germ and radicle of triticale seeds cultured in Petri dish for 7 days.

**Figure 3:**
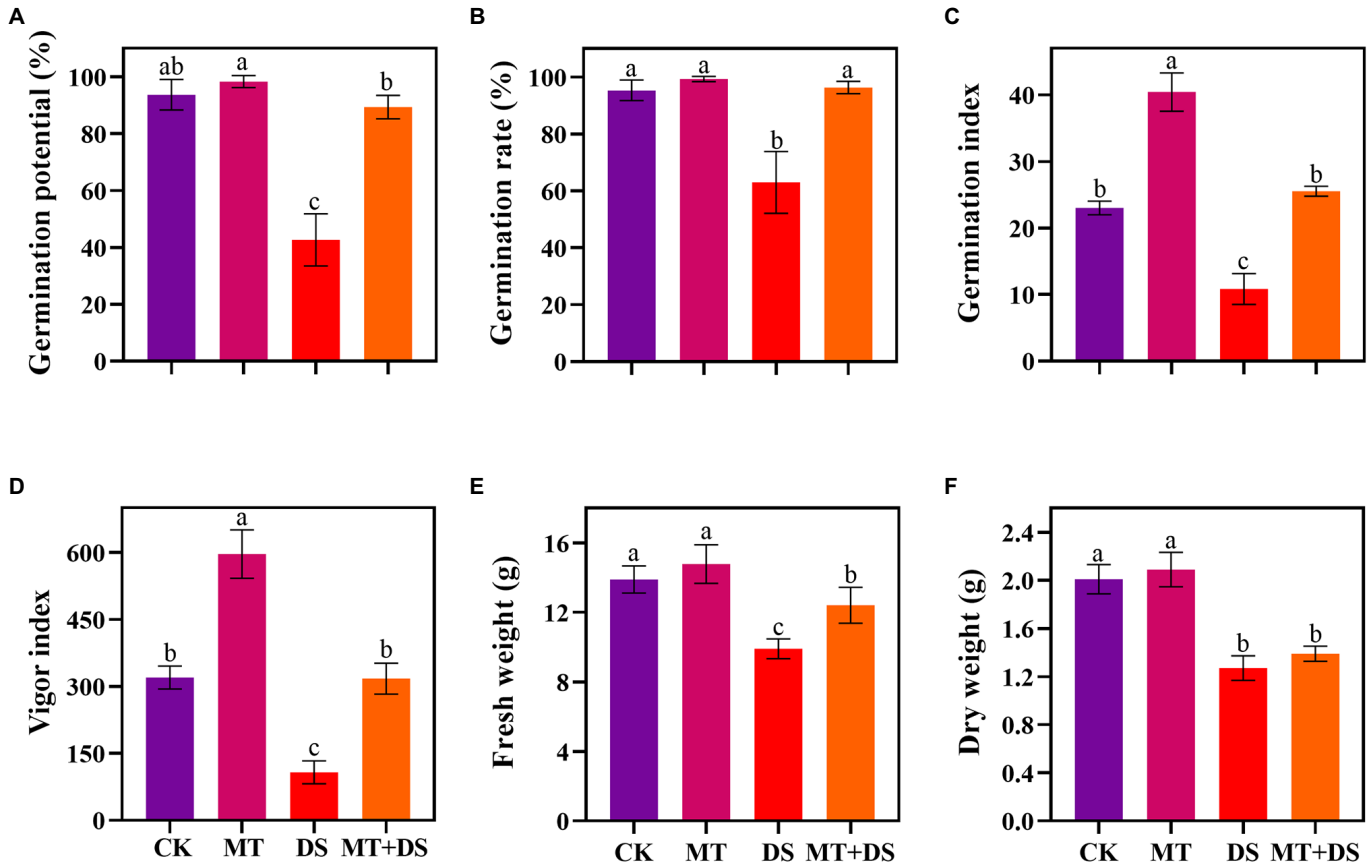
MT at 20 μM concentration was used as priming treatment (MT), 0 μM MT as normal treatment (CK), 10% PEG as drought treatment (DS), and 20 μM MT + 10% PEG treatment (MT+DS). **(A)** Germination potential (GP), **(B)** germination rate (GR), **(C)** germination index (GI), **(D)** vigor index (VI), **(E)** fresh weight, and **(F)** dry weight. Depicted are the means of 6 replicates ± standard error. For each trait, bars with the same letter are not significantly different (*p* < 0.05). Different lower letters indicate significant differences among the treatments (*p* < 0.05).

Drought stress significantly inhibited the germination of triticale seeds. Compared with CK, the length of germ and radicle was significantly shortened by drought stress (Figure 2). Also, the GP, GR, GI, VI, fresh weight, and dry weight decreased by 119.53, 51.32, 112.28, 197.20, 40.19, and 57.90% (Figure 3), respectively, relative to CK. Melatonin priming alleviated the adverse effects of drought stress on seed germination. Compared with DS, GP, GR, GI, VI, fresh weight and dry weight of MT+DS increased by 109.38, 52.91, 135.53, 195.07, 25.25, and 9.67%, respectively. These results indicate that MT priming can markedly improve the seed germination ability under DS.

### Effect of MT Priming on Photosynthesis and Plant Growth

**Figure 4:**
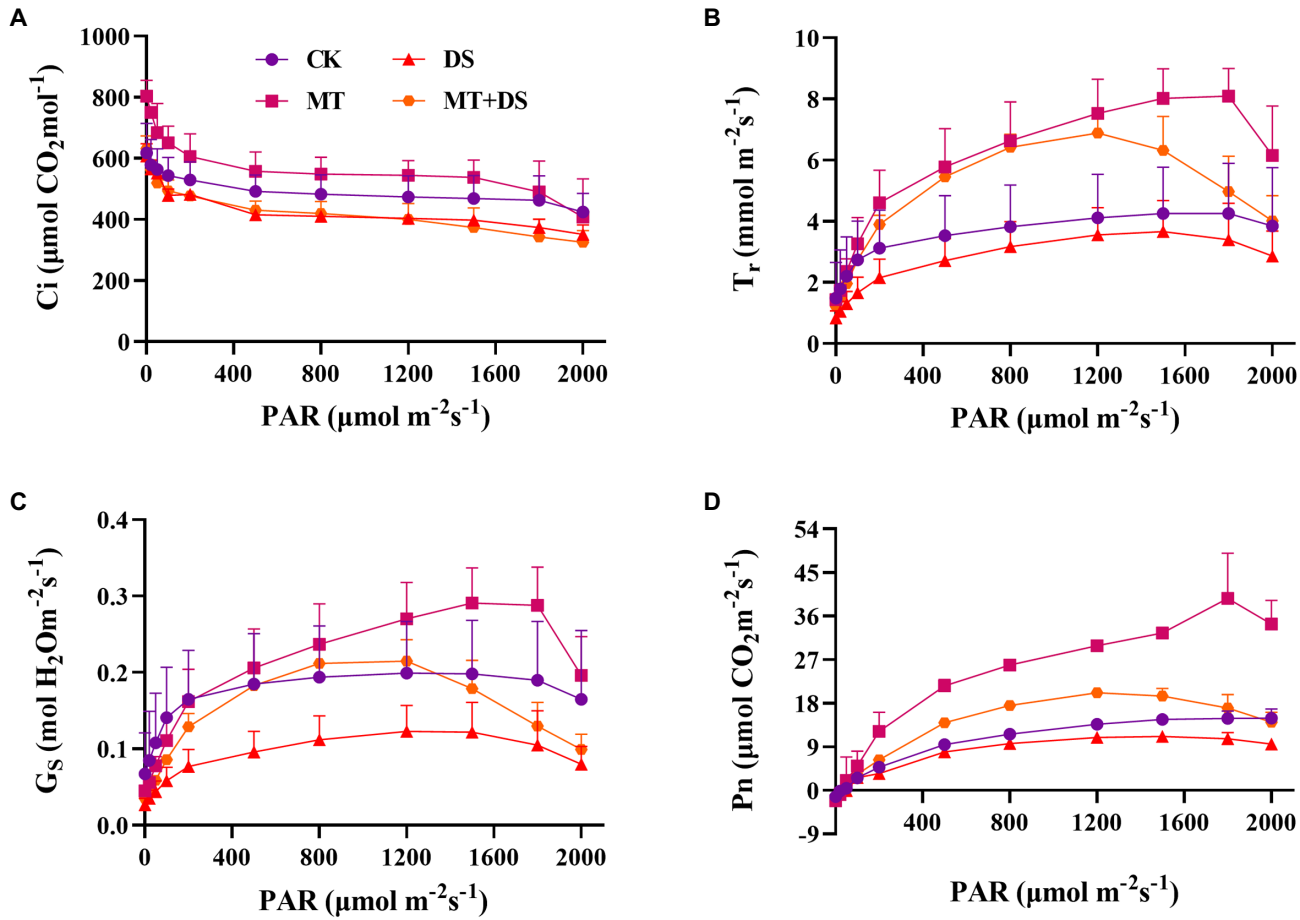
Effects of MT priming on triticale establishment **(A)** intercellular CO_2_ concentration(Ci), **(B)** transpiration rate (Tr), **(C)** stomatal conductance (Gs), and **(D)** net photosynthetic rate (Pn) on light response curves of PAR on 20^th^ day. Depicted are the means of 3 replicates ± standard error.

Drought stress reduced the photosynthetic rate of triticale leaves. As shown in Figure 4D, the Pn, Tr, and Gs all increased with the increase in photosynthetically active radiation (PAR). The relationship between the treatments was as follows: MT>MT+DS>CK>DS. Photosuppression began when the PAR was greater than 1,200 μmol·m^−2^ s^−1^ under drought stress, and when the PAR was greater than 1800 μmol·m^−2^ s^−1^ under normal conditions (Figures 4B–D). During the light response, the Ci decreased significantly when the PAR was less than 100 μmol·m^−2^ s^−1^, and decreased slightly when the PAR was more than 100 μmol·m^−2^ s^−1^ (Figure 4A).

The light saturation point was different in each treatment (Table 1), the MT treatment was 2204 μmol·m^−2^ s^−1^. AQE, Pn_max_, Lcp, Lsp and Pn_max_ were higher in the MT + DS treatment than in the DS treatment.

**Table 1:** Simulation parameters of light response curve of triticale under different treatments for 20 days.

Treatments	LCP (μmol m^−2^ s^−1^)	LSP (μmol m^−2^ s^−1^)	Rd (μmol CO_2_ m^−2^ s^−1^)	Pn_max_ (μmol CO_2_ m^−2^ s^−1^)	AQE (μmol CO_2_ m^−2^ s^−1^/μmol m^−2^ s^−1^)	*R*^2^ of Model fitting
CK	28	2,188	−2.068	19.5053	0.051	0.8629
MT	36	2,204	−2.174	40.6464	0.059	0.8850
DS	32	1,016	−1.004	11.7901	0.031	0.7543
MT + DS	36	1,151	−2.068	20.6885	0.059	0.6654

MT at 20 μM concentration was used as the optimal concentration for priming triticale seeds, unprimed + 70–75% relative water content as CK, primed + 70–75% relative water content as MT, unprimed + 40–45% relative water content as DS, and primed + 40–45% relative water content as MT + DS.

**Figure 5:**
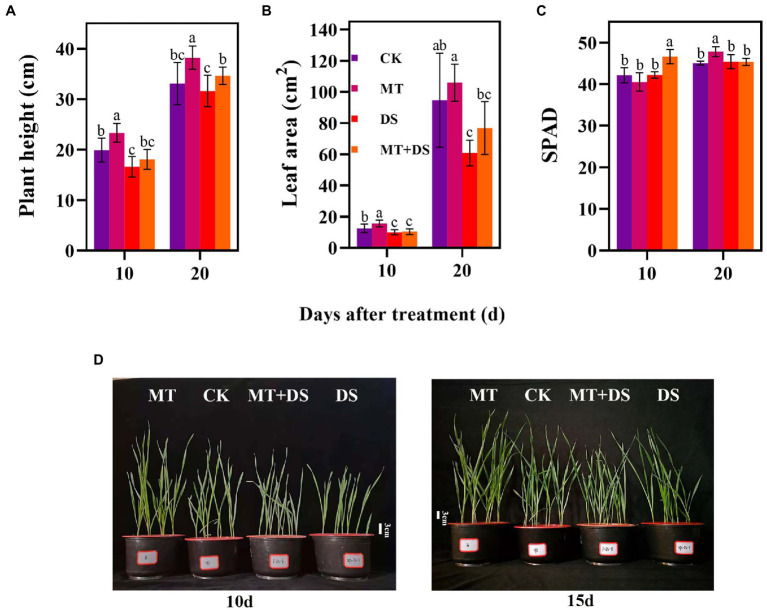
MT at 20 μM concentration was used as the optimal concentration for priming triticale seeds, unprimed +70–75% relative water content as CK, primed +70–75% relative water content as MT, unprimed +40–45% relative water content as DS, and primed +40–45% relative water content as MT+DS. **(A)** Plant height (PHT), **(B)** leaf area (LEA), **(C)** SPAD (relative chlorophyll content) values on the 10^th^ and 20^th^ d after cultivation, and **(D)** phenotypic traits of triticale seedling in pot cultivation for 10^th^ and 15^th^. Depicted are the means of 10 replicates ± standard error. For each trait, bars with the same letter are not significantly different (*p* < 0.05). Different lower letters indicate significant differences among the treatments (*p* < 0.05).

Compared with the CK, the MT treatment increased LEA by 25.23% at 10 d after sowing (Figure 5B). At 20 d after sowing, PHT of MT+DS increased by 8.72% compared with DS treatment (Figure 5A). SPAD of MT treatment was 6.15% higher than that of CK treatment (Figure 5C). Collectively, these results imply that MT priming plays a positive role in promoting photosynthesis and plant growth under drought stress.

### Effect of MT Priming on Root System Characteristics

**Figure 6:**
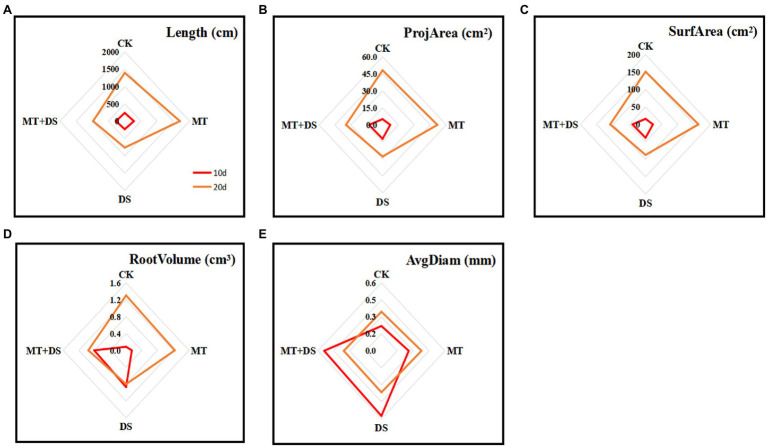
MT at 20 μM concentration was used as the optimal priming concentration for triticale seeds, unprimed +70–75% relative water content as CK, primed +70–75% relative water content as MT, unprimed +40–45% relative water content as DS, and primed +40–45% relative water content as MT+DS. **(A)** Root length, **(B)** root projarea, **(C)** root surface area, **(D)** root volume, and **(E)** root avgdiam of triticale potted seedlings on days 10 and 20.

MT priming increased all the root growth parameters tested in this study. At 20 d after sowing, the root length, root projarea, root surface area, and root volume all increased as follows: MT>CK>MT+DS>DS (Figure 6). At 10 and 20 d after sowing, the root length of MT treatment group increased by 35.64 and 22.18%, respectively, compared with CK. Meanwhile, the root length of MT+DS treatment group increased by 36.34 and 28.23%, and the root volume increased by 21.56 and 19.86% at 10 and 20 d, respectively, compared with DS treatment group. Also, the root surface area of MT+DS treatment group increased by 28.15 and 24.18% at 10 and 20 d, respectively, compared with the DS treatment group.

### Effect of MT Priming on Antioxidant Enzyme Activity

**Figure 7:**
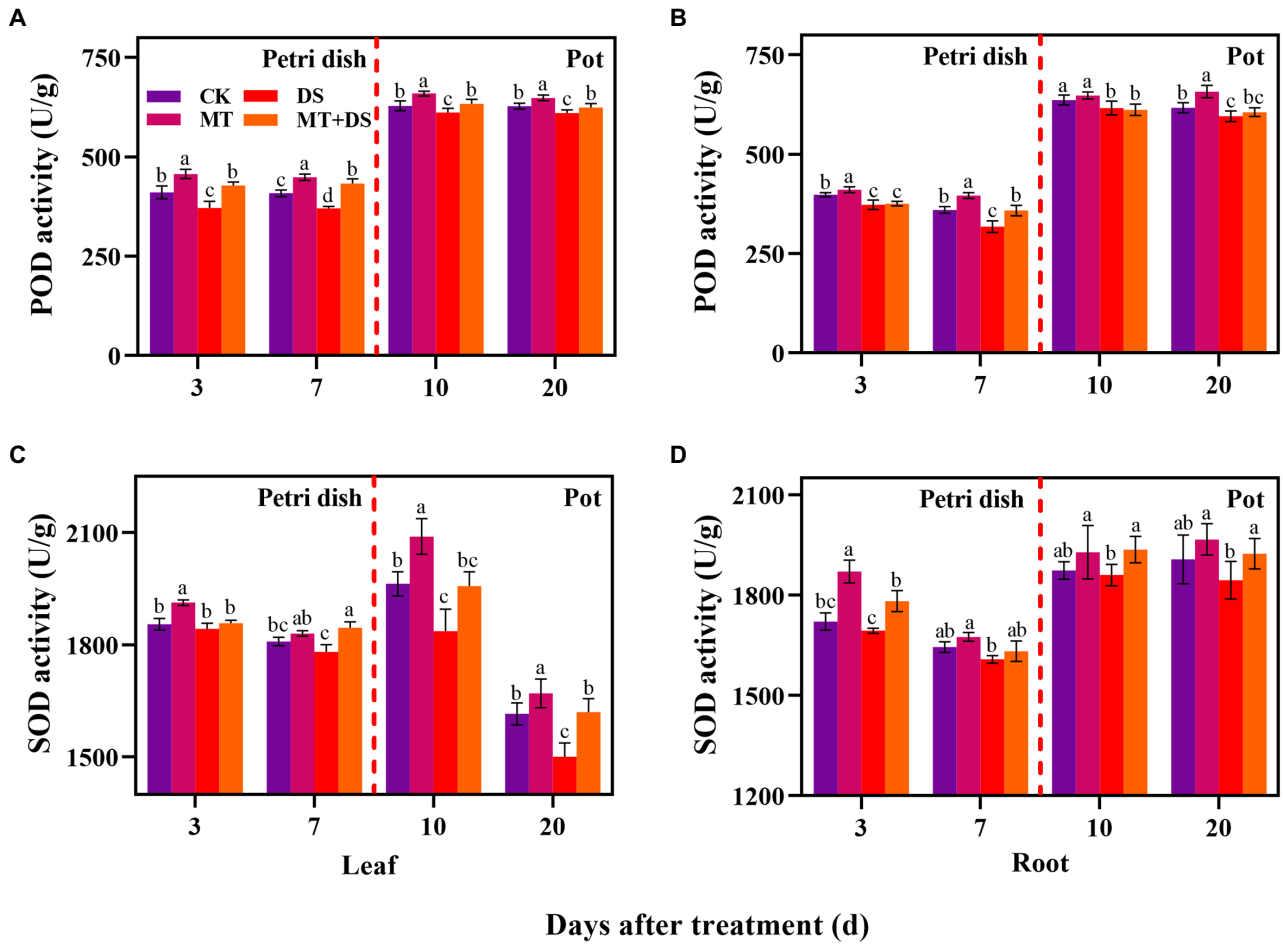
In Petri dish, 20 μM MT was used as the optimal priming treatment for triticale seeds priming (MT), 0 μM MT as normal treatment (CK), 10% PEG as drought treatment (DS), and 20 μM MT + 10% PEG treatment (MT+DS). In pot, 20 μM MT was used as the optimal priming treatment for triticale seedlings, unprimed +70–75% relative water content as CK, primed +70–75% relative water content as MT, unprimed +40–45% relative water content as DS, and primed +40–45% relative water content as MT+DS. The contents of POD **(A)** and **(B)**, SOD **(C)** and **(D)** in leaves and roots of triticale seeds germinated in Petri dishes at 3 and 7 days and seedlings planted at 10 and 20 d. For each trait, bars with the same letter are not significantly different (*p* < 0.05). Different lower letters indicate that there are significant differences in all concentration treatments (*p* < 0.05). Data shown are the averages ± SE (*n* = 6 in A and B; *n* = 3 for Petri dish in **C,D**, *n* = 6 for pot in **C,D**).

The activity of POD in the leaves and roots of triticale seedlings was higher than in the seeds (germ and radicle) under MT treatment. DS treatment reduced the activities of POD and SOD in the germs, radicles, leaves, and roots (Figure 7). Compared with DS, the activity of POD in germs under MT+DS treatment increased by 15.26 and 16.95% at 3 and 7 d after sowing, and by 3.60 and 2.23% in the leaves at 10 and 20 d after sowing, respectively (Figure 7A). In pot experiment, MT+DS treatment increased the SOD activity in the roots by 4.10 and 4.30% at 10 and 20 d after sowing, respectively, compared with DS treatment (Figure 7D). At 3, 7, 10, and 20 d after sowing, the activity of POD under MT treatment increased by 10.24, 24.79, 5.12, and 10.50%, respectively, compared with DS treatment (Figure 7B). Meanwhile, MT treatment increased SOD activity by 3.81, 2.74, 13.79, and 11.28% at 3, 7, 10, and 20 d after sowing, respectively, relative to DS treatment (Figure 7C). Altogether, these findings suggest that MT priming has significant growth repair effect under drought stress.

### Effect of Melatonin Priming on MDA Content

**Figure 8:**
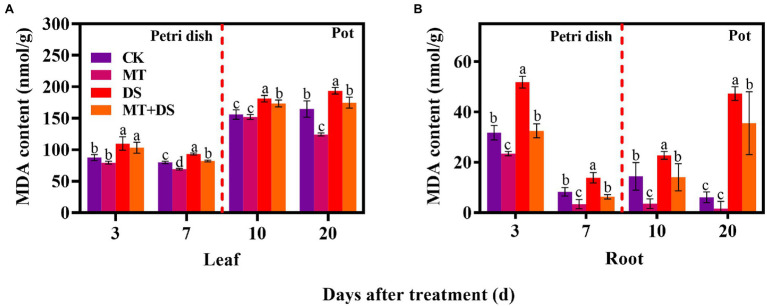
In Petri dish, 20 μM MT was used as the optimal priming treatment for triticale seeds priming (MT), 0 μM MT as normal treatment (CK), 10% PEG as drought treatment (DS), and 20 μM MT + 10% PEG treatment (MT+DS). In pot, 20 μM MT was used as the optimal priming treatment for triticale seedlings, unprimed +70–75% relative water content as CK, primed +70–75% relative water content as MT, unprimed +40–45% relative water content as DS, and primed +40–45% relative water content as MT+DS. **(A)** MDA content in triticale seeds germinated in Petri dishes on 3^rd^ and 7^th^ days and potted seeding leaves on 10^th^ and 20^th^ d; and **(B)** MDA content in radicle of triticale seeds germinated in Petri dish on 3^rd^ and 7^th^ days and seedling roots on 10^th^ and 20^th^ days. For each trait, bars with the same letter are not significantly different (*p* < 0.05). Different lower letters indicate significant differences among the treatments (*p* < 0.05). Data shown are averages ± SE (*n* = 6 in **A,B**).

DS treatment significantly increased the content of MDA in triticale tissues, reflecting cell membrane damage. MT+DS treatment significantly reduced the content of MDA in seeds and seedlings by 37.28, 54.55, 38.09, and 24.08% at 3, 7, 10, and 20 d after sowing, respectively, compared with DS treatment. This alleviated excess oxidation of membrane lipid, reducing the degree of oxidative damage (Figure 8). Meanwhile, MT+DS treatment decreased the MDA content of germ and radicle by 20.59 and 80.58%, respectively, on day seven after sowing, compared to the levels on day three after sowing (Figures 8A,B).

Notably, the MDA content of triticale seedlings (leaf and root) increased continuously as the drought stress continued. Compared with the CK, the MDA content in the roots of the DS treatment increased by 63.07, 67.39, 57.50, and 670.51% at 3, 7, 10, and 20 d after sowing, respectively. MT priming significantly decreased MDA content in seedlings under drought stress (Figures 8A,B). Collectively, these results show that MT priming significantly assuages the damaging effects of drought stress.

### Effect of Melatonin Seed Priming on H_2_O_2_ Accumulation Under Drought Stress

**Figure 9:**
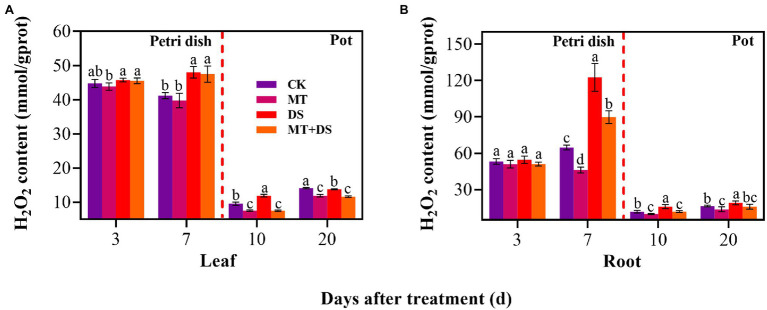
In Petri dish, 20 μM MT was used as the optimal priming treatment for triticale seeds priming (MT), 0 μM MT as normal treatment (CK), 10% PEG as drought treatment (DS), and 20 μM MT + 10% PEG treatment (MT+DS). In pot, 20 μM MT was used as the optimal priming treatment for triticale seedlings, unprimed +70–75% relative water content as CK, primed +70–75% relative water content as MT, unprimed +40–45% relative water content as DS, and primed +40–45% relative water content as MT+DS. **(A)** H_2_O_2_ content of triticale seeds germinated in Petri dishes on 3^rd^ and 7^th^ days and potted seeding leaves on 10^th^ and 20^th^ days; and **(B)** H_2_O_2_ content in radicle of triticale seeds germinated in Petri dish on 3^rd^ and 7^th^ d and seedling roots on 10^th^ and 20^th^ days. For each trait, bars with the same letter are not significantly different (*p* < 0.05). Different lower letters indicate significant differences among the treatments (*p* < 0.05). Data shown are the averages ± SE (*n* = 6 in **A,B**).

MT+DS treatment significantly reduced H_2_O_2_ accumulation in triticale seedlings compared with DS treatment (Figure 9). At 10 and 20 d after sowing, H_2_O_2_ content of the seedlings leaves under MT+DS treatment group decreased by 37.14 and 15.59%, respectively, compared with DS treatment, while in the roots, the content decreased by 25.38 and 16.68% at 10 and 20 d, respectively. Notably, the H_2_O_2_ content was lowest in the seedlings at 10 d after sowing (Figures 9A,B). These results suggest that MT priming reduces H_2_O_2_ accumulation during seedling growth, thus improving seed vigor by suppressing the negative effects caused by reactive oxygen species (ROS) burst.

### Effect of Melatonin Seed Priming on O_2_^−^ Accumulation

**Figure 10:**
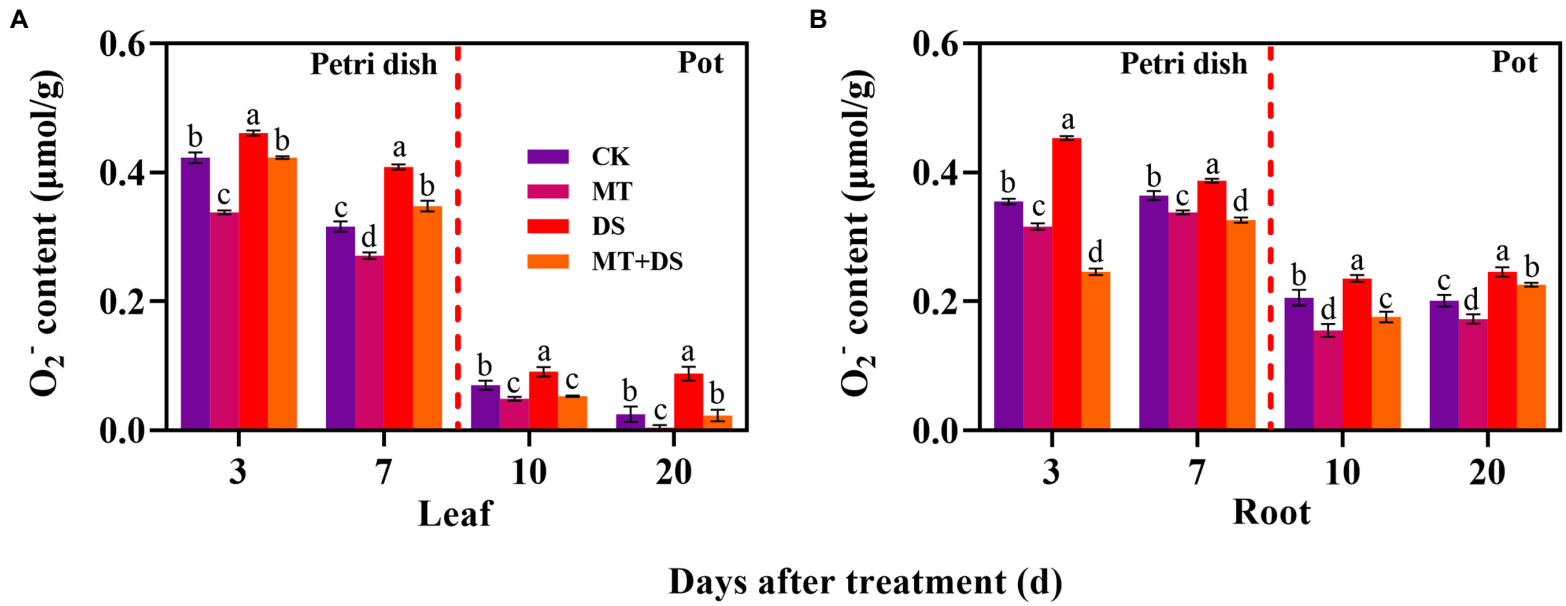
In Petri dish, 20 μM MT was used as the optimal priming treatment for triticale seeds priming (MT), 0 μM MT as normal treatment (CK), 10% PEG as drought treatment (DS), and 20 μM MT + 10% PEG treatment (MT+DS). In pot, 20 μM MT was used as the optimal priming treatment for triticale seedlings, unprimed +70–75% relative water content as CK, primed +70–75% relative water content as MT, unprimed +40–45% relative water content as DS, and primed +40–45% relative water content as MT+DS. **(A)** O_2_^−^ content of triticale seeds germinated in Petri dishes on 3^rd^ and 7^th^ days and potted seedling leaves on 10^th^ and 20^th^; and **(B)** O_2_^−^ content in radicle of triticale seeds germinated in Petri dish on 3^rd^ and 7^th^ day and seedling roots on 10^th^ and 20^th^ d. For each trait, bars with the same letter are not significantly different (*p* < 0.05). Different lower letters indicate significant differences among the treatments (*p* < 0.05). Data shown are the averages ± SE (*n* = 6 in **A,B**).

DS treatment significantly increased the production of O_2_^−^ in triticale seedlings. In contrast, MT treatment significantly inhibited the production of O_2_^−^ in the seedlings. Notably, MT+DS treatment significantly inhibited O_2_^−^ accumulation in the triticale seedlings under drought conditions (Figure 10). At 3, 7, 10, and 20 d after sowing, O_2_^−^ content of MT+DS treatment group decreased by 8.21, 14.95, 41.17, and 73.75%, respectively, compared to DS treatment group. Meanwhile, O_2_^−^ content of DS treatment group was 8.94, 29.65, 29.56, and 253.60% higher than that of CK treatment group at 3, 7, 10, and 20 d after sowing, respectively (Figure 10A). MT+DS treatment decreased O_2_^−^ accumulation in the radicles and roots by 45.56, 15.82, 25.21, and 8.23% at 3, 7, 10, and 20 d after sowing, respectively, compared with DS treatment. The content of O_2_^−^ in the radicles and roots of DS treated seedlings was 27.61, 6.18, 14.42, and 22.67% higher than that of CK treatment group at 3, 7, 10, and 20 d after sowing, respectively, significantly reducing the vigor of triticale seedlings (Figure 10B). Therefore, MT mainly promoted germination and seedling growth under drought stress by increasing the scavenging efficiency of ROS to resist the adverse effects of drought stress.

### Correlation and Principal Component Analyses

**Figure 11:**
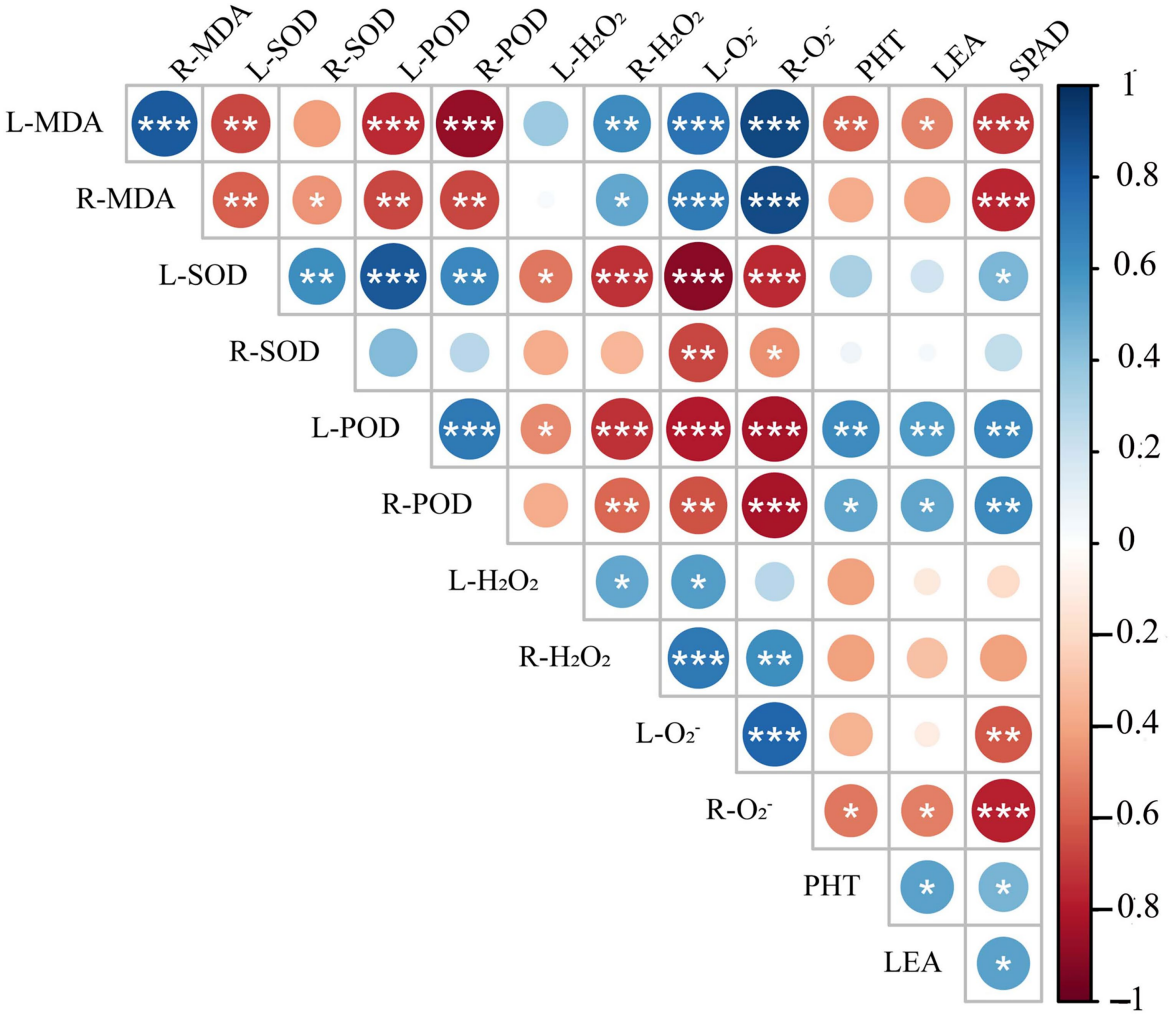
Correlation analysis of 13 indexes between morphological and physiological indicators in triticale seedlings. L-MDA, leaf malondialdehyde content; R-MDA, roots malondialdehyde content; L-SOD, leaves superoxide dismutase activity; R-SOD, roots superoxide dismutase activity; L-POD, leaves peroxidase activity; R-POD, roots peroxidase activity; L-H_2_O_2_, leaves hydrogen peroxide activity; R-H_2_O_2_, roots hydrogen peroxide activity; L-O_2_^−^, leaves superoxide anion activity; R-O_2_^−^, roots superoxide anion activity; PHT, plant height; LEA, leaf area; SPAD, relative chlorophyll content. The asterisk indicates significant difference. ^*^p < 0.05; ^**^p < 0.01; ^***^p < 0.001.

Spearman correlation analysis showed that leaf peroxidase activity (L-POD) was significantly positively correlated with root peroxidase activity (R-POD), PHT, SPAD, and LEA, but significantly negatively correlated with leaf hydrogen peroxide activity (L-H_2_O_2_), root hydrogen peroxide activity (R-H_2_O_2_), leaf superoxide anion activity (L-O_2_^−^) and root superoxide anion activity (R-O_2_^−^) (Figure 11). Leaf superoxide dismutase activity (L-SOD) and root superoxide dismutase activity (R-SOD) were significantly negatively correlated with L-O_2_^−^ and R-O_2_^−^. PHT, LEA, and SPAD showed a significant positive correlation with L-POD, R-POD, a significant negative correlation with leaf malondialdehyde content (L-MDA) and R-O_2_^−^, but no correlation with L-H_2_O_2_, R-H_2_O_2_, R-SOD. These results show that the decrease in plant height and chlorophyll content of triticale seedlings were mainly due to the increase in ROS, H_2_O_2,_ and MDA content, and the decrease in antioxidant enzyme activity.

**Figure 12:**
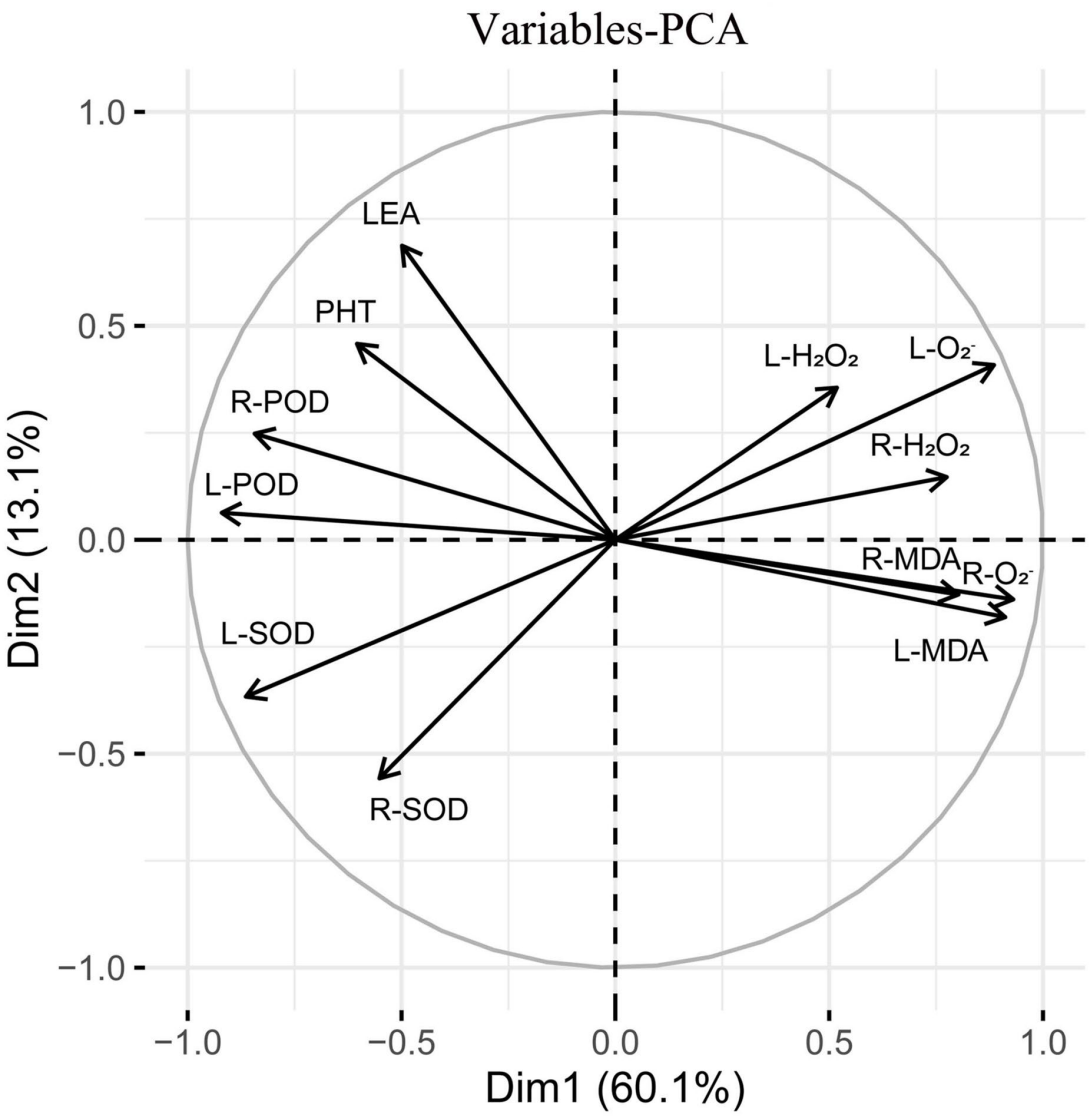
Principal component analysis of 13 indexes (morphology and physiology) in triticale seedlings.

Subsequently, principal component analysis was performed on the triticale seedlings (Figure 12). The contribution rate of the first and second principal components was 73.2% of the total variance. The contribution rate of principal component 1 was relatively large, accounting for 60.1% of the total variance. L-H_2_O_2_, R-H_2_O_2_, L-O_2_^−^, R-O_2_^−^, L-MDA, and root malondialdehyde content (R-MDA) were close to the principal component analysis 1 axis. The contribution rate of principal component 2 was relatively small, accounting for 13.1% of the total variance. L-POD, L-SOD, R-POD, and PHT contributed more to the second principal component.

## Discussion

### MT Priming Promotes Seed Germination Under Drought Stress

Phytohormones such as MT, auxin, GA_3_, cytokinin, and ABA play crucial roles throughout the life cycle of plants (Jiang and Guo, 2010). The seed germination process is coordinated by a variety of hormones (Obroucheva, 2012). In this study, 20 μM MT emerged as the optimal concentration for triticale seed priming under DS. MT at 20 μM promoted seed germination, as exhibited by several seed germination parameters, including higher germination potential, germination rate, germination and vigor indexes, and longer germ and radicle (Figure 1). These results are consistent with previous studies, suggesting that MT priming improves seed germination under drought stress (Heshmati et al., 2021). The mitigating effect of MT on seed germination under stress conditions is closely related to its concentration. For example, Cao et al. (2019) showed that in waxy corn, 50 μM was the optimal concentration for MT priming under low-temperature stress. Khan et al. (2019) concluded that 500 μM was the optimal MT priming concentration under drought stress in rapeseed. Therefore, the optimal MT concentration for seed priming is related to species and environmental conditions (Wen et al., 2016).

Adverse environmental conditions increase the accumulation of ROS (Kaya and Doganlar, 2019). ROS such as (OH^−^) and singlet oxygen (O_2_^−^) are hydroxyl radicals whose accumulation can aggravate membrane lipid peroxidation (Del Río, 2015). MT priming can reduce the accumulation of ROS in seeds (Zhang et al., 2014). Abiotic stress, especially drought stress can induce ROS burst in plants, leading to increased accumulation of H_2_O_2_ and other hydroxyl radicals, which affect plant growth and development (Lu et al., 2009). SOD and POD are key enzymes of the protective enzyme system and play a pivotal role in scavenging ROS. In this study, MT priming increased the activities of SOD and POD, but decreased the contents of H_2_O_2_, O_2_^−^, and MDA under DS (Figures 7–10). Xiao et al. found that soaking seeds in 20 μM MT solution was optimal for reducing the accumulation of MDA and increasing the activities of SOD and POD under drought stress in cotton (Xiao et al., 2019). Similarly, MT priming alleviated the effect of low-temperature stress on cucumber seed germination by enhancing protective enzyme activity (Posmyk et al., 2009). In the present study, MT priming enhanced ROS scavenging ability of triticale seeds under DS, thus promoting seed germination and growth.

### MT Priming Promotes Seedling Growth Under Drought Stress

Drought stress seriously affects the photosynthetic capacity of crop seedlings (Liu et al., 2015a). Drought has a negative effect on photosynthesis in wheat (Ahmad et al., 2018). Drought stress can also impair photosynthetic activities of plants, leading to severe growth retardation (Altaf et al., 2022). A study reported that Pn of cotton decreased with the increase in drought intensity (Deeba et al., 2012). In maize, Pn decreased depending on the intensity and duration of water stress (Cai et al., 2020). Excessive ROS damages chloroplasts, inhibiting crop photosynthesis and yield (Li et al., 2014). In the present study, MT priming improved photosynthetic performance, especially Pn and Tr (Figures 4B,D), thus increasing the accumulation of photosynthetic products in the triticale seedlings under DS. MT priming can also improve the structure of plants (Tiwari et al., 2021). In a previous study, rapeseed treated with 500 μM MT exhibited longer plant height and higher aboveground biomass under drought stress (Khan et al., 2020). These reports are consistent with our findings, which also showed that MT priming increased the plant height, leaf area, and root length of triticale seedlings under DS (Figures 5, 6).

The root system is the main organ for absorbing water and nutrients in plants, and is the primary part of drought stress response. The root morphology of plants normally changes to maximize the utilization of water hydraulic environment and alleviate the damage caused by drought stress (Vadez, 2014). The root system quickly transmits signals to the leaves after sensing drought stress, changes stomatal shape, and improves water use efficiency (Jiao et al., 2021). Therefore, root traits are closely related to the drought tolerance of crops (Ekanayake et al., 1985; Djanaguiraman et al., 2019; Liang et al., 2019). In this study, MT priming increased the total root length of triticale seedlings by 35.64 and 22.18% at 10 and 20 d after sowing, respectively, compared with CK (Figure 6). Notably, 100 μM MT priming also increased the root growth rate and length in tomato (Liu et al., 2015a). Furthermore, MT has been shown to promote root development under abiotic stresses like salt stress (Guo et al., 2015). In this study, MT treatment increased the root length, root projarea, root surface area, and root volume by 36.34, 28.52, 28.52, and 21.56%, respectively, compared with CK under DS (40–45%). These findings further show that drought stress affects the photosynthetic traits of triticale seedlings, limiting the increase in plant height and root length while, MT priming promotes seedling development, and alleviates the negative effects of drought stress.

**Figure 13:**
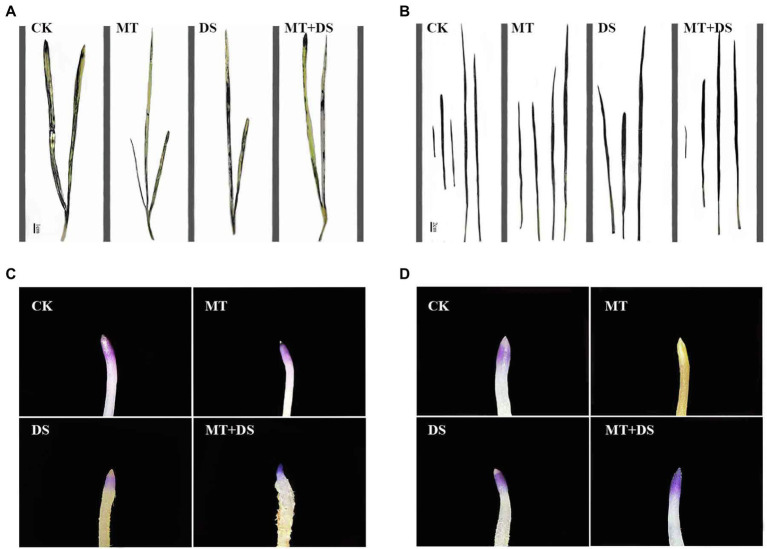
NBT histochemical detection of O_2_^−^ in triticale plant parts. **(A)** Leaf staining on 10^th^ day, **(B)** leaf staining on 20^th^ day, **(C)** root staining on 10^th^ day and **(D)** root staining on 20^th^ day. To visualize the NBT deposits, leaves were decolorized in boiling ethanol and the blue deposits of O_2_^−^ in form of spots were quantified.

**Figure 14:**
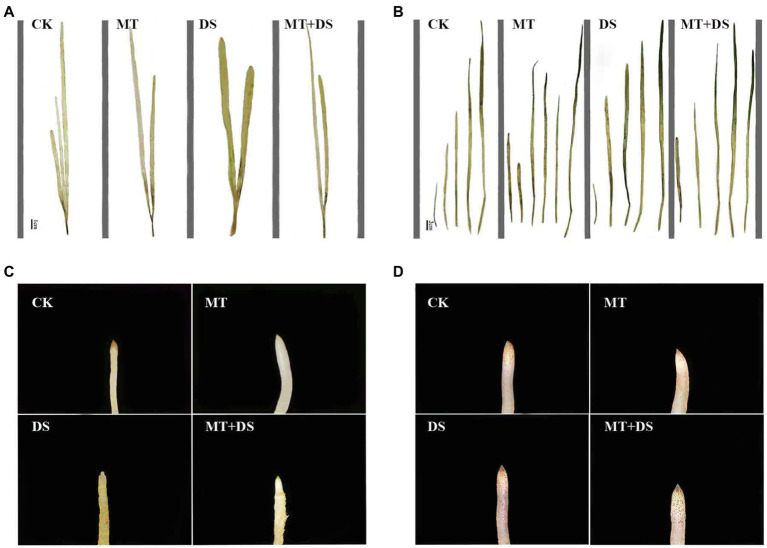
DAB histochemical detection of H_2_O_2_ in triticale plant parts. **(A)** Leaf staining on 10^th^ day, **(B)** leaf staining on 20^th^ day, **(C)** root staining on 10^th^ day and **(D)** root staining on 20^th^ day. To visualize the DAB deposits, leaves were decolorized in boiling ethanol and the brown sediments of H_2_O_2_ in the spots were quantified.

MT treatment enhanced ROS scavenging ability of seedlings (Yu et al., 2018; Sharma and Zheng, 2019), and promoted plant development under drought stress (Moustafa-Farag et al., 2020). MT treatment significantly reduced H_2_O_2_ and O_2_^−^ contents of wheat (500 μM) and cotton (100 μM) seedlings (Cui et al., 2017; Bai et al., 2020). Seed priming with 25 μM MT promoted the scavenging of ROS in cotton seedlings under salt stress (Zhang et al., 2021). Tiwari et al. (2021) found that MT regulates the ascorbic acid-glutathione (AsA-GSH) cycle, which plays a crucial role in scavenging ROS under drought stress. In this study, drought stress increased the H_2_O_2_ and O_2_^−^ contents of triticale seedlings, while MT priming significantly reduced the accumulation of H_2_O_2_ and O_2_^−^ (Figures 13, 14). Specifically, MT priming reduced ROS accumulation in root tips and leaves of triticale seedlings under DS, thereby reducing the degree of tissue damage. Further principal component analysis showed that the H_2_O_2_, O_2_^−^ and MDA contents of leaves and roots were the most responsive to MT priming (Figure 12). Collectively, these findings show that MT priming alleviates adverse effects of drought stress by enhancing ROS scavenging.

ROS scavenging is associated with increased activity of protective enzyme systems. Specifically, SOD and POD are key enzymes in the protective enzyme system, which play a crucial role in scavenging ROS in plants (Li et al., 2017). In the present study, MT treatment increased the activity of POD and SOD significantly, thus improving the ROS removal ability. Similar results were also reported in oilseed (Khan et al., 2019).

MT priming enhances ROS scavenging ability, and reduces MDA accumulation (Altaf et al., 2022). The accumulation of MDA is positively correlated with the degree of cell membrane lipid peroxidation, reflecting the degree of damage to the cell membrane (Imran et al., 2021). Drought stress disrupts the balance between intracellular ROS generation and scavenging, reducing ROS scavenging ability, resulting in MDA accumulation (Li et al., 2019), which accelerates cell membrane system damage. Here, we also found that drought stress increased MDA content in leaves and roots of triticale seedlings, consistent with the results previously reported in wolfberry by Guo et al. (2018). Principal component analysis showed that the MDA content of leaves and roots had a greater response to MT triggering, with individual contribution rate of between 9 and 10% (Figure 12), suggesting that suppression of MDA accumulation is an additional factor by which MT priming alleviates adverse effects of drought stress in plants.

**Figure 15:**
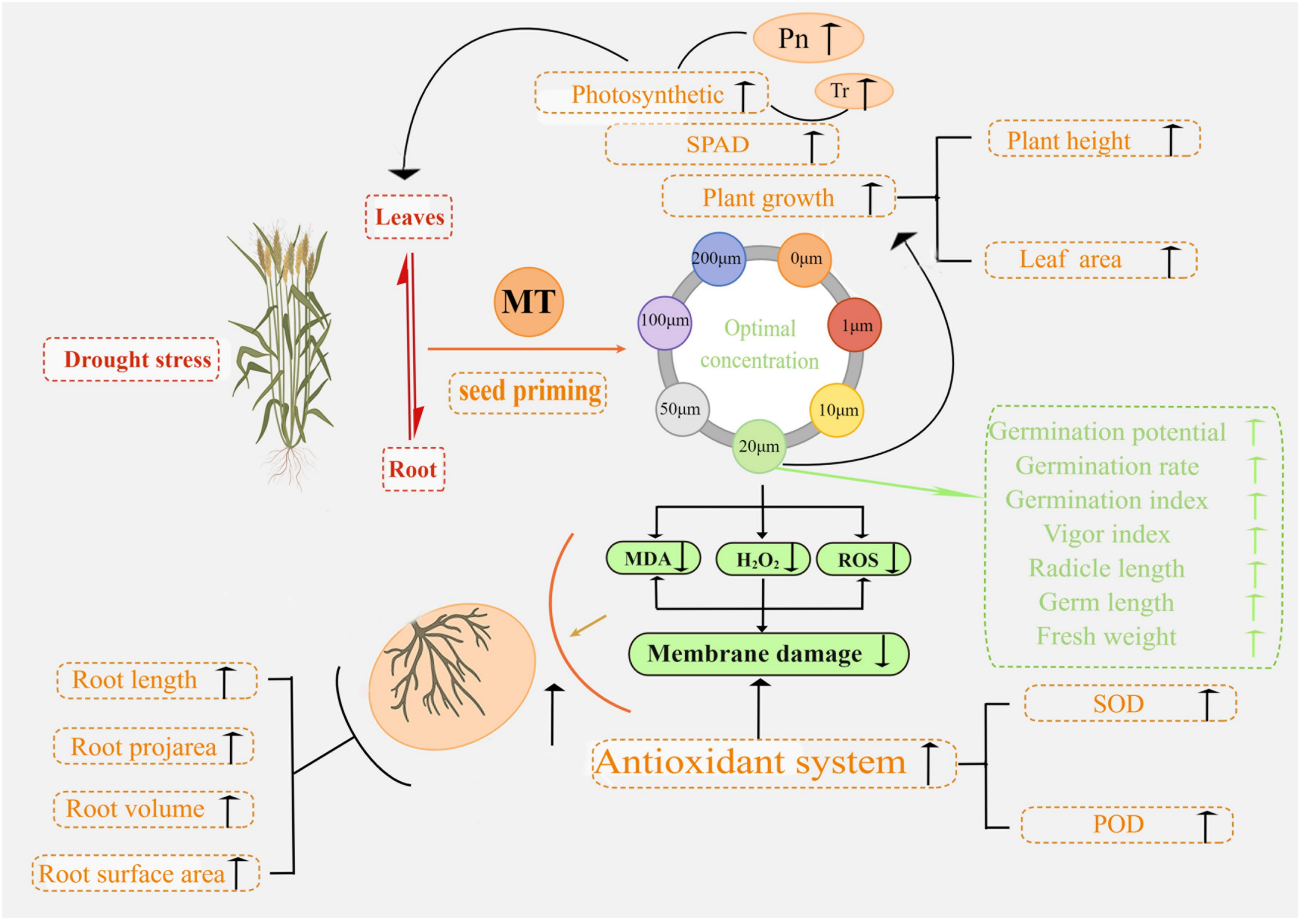
Proposed mechanism of action underlying the enhancement of seed germination and seedling growth by melatonin priming under drought stress.

The mechanisms underlying MT seed priming to promote seed germination and seedling development and improve plant resistance to drought stress in triticale were uncovered in this study. In Figure 15, we summarize the response of triticale seeds and seedlings to drought stress following MT priming based on the morphological and physiological data. This study improves the understanding of MT priming to promote drought tolerance in triticale seeds, providing a new target for improving agronomic management practices.

## Conclusion

Seed priming with MT (20 μM) significantly enhanced triticale seed germination and seedling growth under DS, accelerating antioxidant enzyme activity against ROS accumulation. Specifically, MT priming increased the antioxidant enzyme activities in triticale seeds (germ and radicle) and seedlings (leaf and root), enhanced the scavenging ability of ROS, and decreased the contents of MDA, H_2_O_2_, and O_2_^−^, thus reducing the damage caused by oxidative stress under DS. These findings suggest that seed priming with melatonin promotes ROS scavenging capacity, and enhances energy supply and antioxidant enzyme activities under DS, thereby alleviating the adverse effects of drought stress in triticale seedlings. To the best of our knowledge, this is the first study to report that melatonin alleviates drought stress in triticale. Seed priming with meltonin is a promising approach for enhancing seed germination and seedling growth under DS. However, further studies should be conducted under field conditions to explore the applicability of our findings.

## Data Availability Statement

The raw data supporting the conclusions of this article will be made available by the authors, without undue reservation.

## Author Contributions

YG, LL, HD, LT, and CL: conceived the study and proposed the methods. YG, LL, DL, HS, LZ, KZ, and HZ: contributed to the preparation of equipment and acquisition of data. DL, YZ, AL, ZB, and CL: validated results. YG: wrote the paper. LL, LT, HD, and CL: revised the paper. All authors contributed to the article and approved the submitted version.

## Funding

This study was supported by grants from the National Natural Science Foundation of China (No. 31871569 and 32172120).

## Conflict of Interest

The authors declare that the research was conducted in the absence of any commercial or financial relationships that could be construed as a potential conflict of interest.

## Publisher’s Note

All claims expressed in this article are solely those of the authors and do not necessarily represent those of their affiliated organizations, or those of the publisher, the editors and the reviewers. Any product that may be evaluated in this article, or claim that may be made by its manufacturer, is not guaranteed or endorsed by the publisher.
